# Differential Virulence of Vaginal *Candida albicans* Isolates Correlates with Host Inflammatory Responses in VVC/RVVC

**DOI:** 10.3390/jof12070509

**Published:** 2026-07-10

**Authors:** Natalia Pedretti, Luca Spaggiari, Francesco Ricchi, Samyr Kenno, Muhammad Behzad, Samuele Peppoloni, Karin Sossi, Giuseppina Campisciano, Andrea Ardizzoni, Francesco De Seta, Manola Comar, Eva Pericolini

**Affiliations:** 1 Hiptech PhD Program, University of Modena and Reggio Emilia, 41125 Modena, Italy; natalia.pedretti@unimore.it (N.P.); muhammad.behzad@unimore.it (M.B.); 2 Department of Surgical, Medical, Dental and Morphological Sciences with Interest in Transplant, Oncological and Regenerative Medicine, University of Modena and Reggio Emilia, 41125 Modena, Italy; luca.spaggiari@unimore.it (L.S.); francesco.ricchi@unimore.it (F.R.); samyr.kenno@unimore.it (S.K.); samuele.peppoloni@unimore.it (S.P.); andrea.ardizzoni@unimore.it (A.A.); 3 Department of Advanced Translational Microbiology, Institute for Maternal and Child Health-IRCCS Burlo Garofolo, Via dell ’Istria 65/1, 34137 Trieste, Italy; karin.sossi@burlo.trieste.it (K.S.); giusi.campisciano@burlo.trieste.it (G.C.); manola.comar@burlo.trieste.it (M.C.); 4 Department of Obstetrics and Gynecology, IRCCS San Raffaele Scientific Institute, University Vita and Salute, 20132 Milan, Italy; fradeseta@gmail.com; 5 Department of Medicine, Surgery and Health Sciences, University of Trieste, Strada di Fiume 447, 34149 Trieste, Italy

**Keywords:** *Candida albicans*, vulvovaginal candidiasis, vaginal epithelial cells, vaginal microbiota, mucosal immunity

## Abstract

*Candida albicans* (*C. albicans*) is a commensal of the vaginal mucosa and the main etiological agent of acute and recurrent vulvovaginal candidiasis (VVC/RVVC). Disease severity is thought to depend on a dysregulated host inflammatory response to *Candida*, not necessarily associated with increased fungal burden and/or morphogenesis. The role of strain-specific differences leading to epithelial immune response or tolerance remains undefined. In this study, we compared the virulence profile of vaginal *C. albicans* isolates from women with acute VVC/RVVC (VVC/RVVC), asymptomatic colonizer (Colonizing), and VVC/RVVC associated with microbial co-infections (Co-infections). Isolates were evaluated for growth and biofilm formation under standard culture conditions and tested in an *in vitro* vaginal epithelial cell (VEC) infection model to assess fungal shedding, epithelial damage, and cytokine production. Corresponding vaginal samples were analyzed for *C. albicans* morphology, polymorphonuclear neutrophil presence, microbiota composition, cytokines levels, and anti-*C. albicans* IgA production. No significant differences in growth or biofilm formation were observed among isolates under culture conditions. However, VEC infection revealed strain-dependent differences: acute VVC/RVVC and Co-infections isolates induced greater fungal shedding, while VVC/RVVC isolates caused increased epithelial damage and showed a trend toward higher cytokine production. Vaginal samples from symptomatic groups displayed increased neutrophils, hyphal morphology, elevated IL-1α, IL-1β, and anti-*Candida* IgA, but not IL-1Ra, without differences in lactobacilli abundance or Community-State-Type (CST) distribution. These findings suggest that *C. albicans* pathogenicity in VVC depends on strain-specific interactions with VEC driving differential host responses.

## 1. Introduction

Vulvovaginal candidiasis (VVC) is one of the most common mucosal fungal infections affecting women of reproductive age. It is estimated that approximately 75% of women experience at least one episode during their lifetime, while 5–8% develop recurrent vulvovaginal candidiasis (RVVC). The recurrent infection poses a significant clinical burden and is associated with impaired quality of life [1,2]. Despite the availability of anti-fungal treatments, high recurrence rates persist, indicating the limitations of current therapeutic approaches [3].

Most cases of VVC are caused by *C. albicans*, a dimorphic opportunistic fungus that normally resides as a commensal organism within the vaginal mucosa. However, under certain host- and environmental-related conditions, *C. albicans* can undergo a transition from a harmless commensal to a pathogen [1,4]. Increasing evidence suggests that the pathogenesis of VVC is not solely driven by fungal overgrowth and yeast-to-hyphae transition [5]; rather, disease severity is largely determined by the exacerbation of the host inflammatory response to the fungus [3,4]. Therefore, VVC is recognized as a predominantly immunopathological disorder. Indeed, aberrant activation of vaginal epithelial cells in response to *C. albicans* promotes excessive polymorphonuclear neutrophils (PMN) recruitment, which, rather than contributing to effective fungal clearance, leads to epithelial damage and symptoms [2,6]. This immunopathological model is especially evident in RVVC, where symptomatic episodes often arise despite similar levels of vaginal colonization, indicating dysregulated interactions between host and fungus [7].

At the fungal level, a key determinant of *C. albicans* pathogenicity in the vaginal niche is its ability to undergo dimorphic transition. Notably, the yeast-to-hypha transition is closely linked to epithelial invasion and subsequent activation of host immune responses [1,8]. Hyphal growth enables the expression of virulence factors, including candidalysin, a peptide toxin that induces epithelial damage and activates cell danger-associated signaling pathways [9]. In the vaginal epithelial cells’ infection model, candidalysin-mediated injury is accompanied by mitochondrial activation and increased production of reactive oxygen species, which contributes to amplifying inflammatory responses [9].

Beyond hyphal transition, disease pathogenesis seems to be influenced by strain-specific differences among clinical isolates of *C. albicans*. Indeed, *in vitro* comparative studies have demonstrated that vaginal fungal isolates obtained from women with VVC induce higher epithelial damage, elicit stronger pro-inflammatory responses, and promote increased fungal shedding; the latter consists in the release of fungal cells attached to exfoliated epithelial cells from the infected mucosal surface [7,10]. Taken together, these findings underscore the role of fungal heterogeneity in determining clinical manifestations and in increasing the risk of recurrence.

Recent studies have identified specific fungal proteins that exacerbate vaginal inflammation. The pH-regulated antigen Pra1 (a zinc-binding protein), has been recognized as a key immunopathogenic factor in VVC, promoting PMN recruitment and intensifying local inflammatory responses [6]. Notably, inhibiting Pra1 expression through zinc supplementation significantly reduced vaginal inflammation and disease severity in both mice and in women, suggesting a non-anti-fungal host-directed therapeutic strategy for RVVC [6].

Advances in mucosal immunology have broadened the understanding of how epithelial cells respond to *C. albicans* [4,11]. Vaginal epithelial cells activate intracellular immune pathways, including the complosome, in response to fungal challenge. Different clinical isolates of *C. albicans* modify this pathway to varying extents, which may influence epithelial inflammation and disease progression [11].

Overall, current evidence supports a multifactorial model of VVC pathogenesis in which the disease outcome depends on the interplay of strain-specific pathogenicity, host immune responses, and the vaginal microbiota [1,3,4]. To date, the mechanisms explaining how strain-dependent fungal traits integrate with epithelial inflammatory responses and clinical features, particularly in the context of polymicrobial vaginal environments, remain poorly understood. By clarifying these unresolved aspects, we will provide a key to deepen our knowledge of VVC pathogenesis which will allow us to design more effective, personalized, host-directed interventions.

## 2. Materials and Methods

### 2.1. Subject Population

Prior to enrollment, each subject answered a questionnaire reporting their health status and symptoms of vaginal disease and provided informed consent. Subjects with VVC without or with a history of RVVC, defined as at least four acute episodes in one year, were included in the study. For these women, the presence or absence of vulvovaginal signs (congestion, edema, scratches, rhagades, erosions, and secretion volume) and symptoms (vaginal discharge, itching, burning sensation, pain, dryness, and erythema) were assessed. In addition, isolation of *C. albicans* and its determination by the microscopic examination of a wet mount of vaginal sample with potassium hydroxide (KOH) or Gram staining were required. This group of women, the respective vaginal samples and the *C. albicans* isolates were categorized as VVC/RVVC.

Women with concomitant vulvovaginitis or bacterial vaginosis caused by other pathogens (aerobic or anaerobic bacteria, respectively), in addition to the presence of *C. albicans* and vulvovaginal signs and symptoms, the respective vaginal samples and the *C. albicans* isolates, were categorized as Co-infections. Finally, healthy subjects without signs and symptoms of vulvovaginal disease, but positive for the presence of *C. albicans*, the respective vaginal samples and the *C. albicans* isolates, were categorized as Colonizing.

The study was approved by the Institutional Review Board of the IRCCS Burlo Garofolo, Trieste, Italy (IRB-BURLO 03/2023 27.04.2023) and all the experiments were conducted according to the principles stated in the Declaration of Helsinki.

### 2.2. Vaginal Clinical Samples Collection

Vaginal samples were collected using a sterile swab with liquid transport medium (cliniswab DS 321/SG, APTACA, Canelli, Italy) by performing a single gentle 360° rotation of the swab at the vaginal wall. Upon arrival at the microbiology laboratory, vaginal swabs were resuspended in sterile saline solution (0.9% NaCl) to obtain homogenized clinical samples. Each vaginal sample was seeded onto *Candida* chromogenic agar (CAN2) (bioMérieux, Marcy-l’Étoile, France) and incubated at 37 °C for 24–48 h. After visible growth, *Candida* colonies were used to prepare glycerol stocks in a 50:50 mixture of Brain Heart Infusion (BHI) broth and sterile glycerol (Oxoid, Milan, Italy). The glycerol stocks were then stored at −80 °C for long-term preservation. Species-level identification of *Candida* isolates was performed using the Allplex™ Candidiasis Assay (Seegene, Seoul, South Korea), according to the manufacturer’s instructions. Vaginal wet mount preparations from vaginal samples were examined to assess the presence of PMN and hyphae. Based on microscopic observation the samples were categorized as positive or negative for hyphal and PMN presence.

Part of the vaginal samples were also used for Next Generation Sequencing (NGS) analysis and Community-State-Type (CST) categorization and for cytokine determination, as described below.

### 2.3. C. albicans Strains and Culture Conditions

Clinical *C. albicans* isolates used in this study were provided by the IRCCS Maternal and Child Health Institute Burlo Garofolo (Trieste, Italy). A total of 25 fungal strains were assessed, including *C. albicans* strains obtained from women with VVC/RVVC (n = 8), colonizing strains from healthy asymptomatic women (n = 7), and strains collected from patients with RVVC and concomitant bacterial co-infections (n = 10). All fungal isolates were stored at −80 °C in cryovials containing plastic beads (Pro-Lab Diagnostics, Bromborough, UK) and maintained by weekly subculturing on Sabouraud Dextrose Agar (SDA) plates (Oxoid, Milan, Italy). For each experiment, a single colony was inoculated into 5 mL of Yeast Extract-Peptone-Dextrose (YPD) broth (Condalab, Madrid, Spain) and incubated at 37 °C with agitation for ~18 h to reach the exponential phase.

### 2.4. Next Generation Sequencing (NGS) Analysis

Bacterial DNA from vaginal samples for microbiome analysis was extracted using the Maxwell CSC Blood DNA Kit for the Maxwell CSC Instrument (Promega, Madison, WI, USA), as indicated by the supplier. All nucleic acids were stored at −80 °C before further manipulation. The vaginal microbiome was profiled by sequencing the V3–V4 regions of the 16S rRNA gene on the MiSeq Illumina Platform, using the Quick-16S NGS Library Prep Kit (Zymo Research, Irvine, CA, USA), following the manufacturer’s instructions. Raw sequencing data were processed using QIIME2 2022-2. Silva v138 was chosen for the taxonomy assignment, with a BLAST+ v2.12.0 consensus approach.

### 2.5. Phenotypic Characterization of C. albicans Clinical Isolates In Vitro: Growth Kinetics and Biofilm Formation

Growth kinetics were assessed for all 25 *C. albicans* strains over a total period of 48 h. A single colony from each strain was inoculated into 5 mL of YPD broth and incubated overnight at 37 °C under agitation. Following incubation, cultures were adjusted to a final concentration of 1.5 × 10^3^ CFU/mL, as previously described [12]. Aliquots of 200 µL were dispensed into a 96-well flat-bottom microtiter plate (Corning Inc., New York, NY, USA) and then incubated into a 96-well microplate reader (Tecan Sunrise™, Tecan Group Ltd., Männedorf, Switzerland). Optical density (OD) was measured at 570 nm every 2 h throughout the incubation period.

Biofilm formation was assessed according to previously established protocol [13]. Briefly, single colonies of clinical isolates grown on SDA plates were inoculated into 5 mL of YPD broth and incubated overnight at 37 °C under agitation. Yeast suspensions were diluted in RPMI 1640 medium (Euroclone, Milan, Italy) supplemented with L-glutamine (SIAL Group, Rome, Italy) and 34.5 g/L MOPS (without sodium bicarbonate; pH 7) to a final concentration of 1 × 10^7^ cells/mL. Aliquots of 200 µL were transferred into each well of a 96-well flat-bottom microtiter plate (Corning Inc., New York, NY, USA). Plates were incubated aerobically at 37 °C for 1.5 h to allow initial adhesion. Following incubation, the supernatant was removed, and wells were gently washed twice with 200 µL of phosphate-buffered saline (PBS; Sigma-Aldrich, St. Louis, MO, USA) to eliminate non-adherent cells. Fresh RPMI 1640 (200 µL) was then added to each well, and plates were further incubated for 24 h under the same conditions to allow biofilm maturation. Biofilm biomass was quantified using the crystal violet (CV) staining assay, as previously described [13]. After incubation, the culture medium was discarded, and plates were air-dried for 45 min. Wells were washed twice with 200 µL PBS and stained with 110 µL of 0.4% CV solution for 45 min. Excess stain was removed by washing the wells four times with 200 µL of distilled water. The CV retained by the biofilm was solubilized by adding 200 µL of 95% ethanol (Carlo Erba Reagents, Milan, Italy), followed by incubation for 45 min. Absorbance was measured at 590 nm using a 96-well microplate reader. The biofilm-forming capacity of each isolate was classified as strong, moderate, weak, or non-biofilm producer based on the criteria described by Stepanović et al. [14].

### 2.6. Human Vaginal Epithelial Cells

The A-431 cell line (ATCC CRL-1555, Manassas, VA, USA) derived from human vaginal squamous cell carcinoma, was used. Vaginal epithelial cells (VECs) were maintained in Dulbecco’s Modified Eagle’s Medium High-Glucose (DMEM) (SIAL Group, Rome, Italy) supplemented with penicillin (100 U/mL), streptomycin (100 µg/mL; Sial S.p.A., Roma, Italy), L-glutamine (200 mM; SIAL Group, Rome, Italy), and heat-inactivated fetal bovine serum (FBS; Sial, Roma, Italy) at a final concentration of 10% for routine culture or 5% during infection assays. Vaginal epithelial cells were incubated at 37 °C in a humidified atmosphere containing 5% CO_2_ and maintained by weekly passages.

To establish a confluent VEC monolayer, the day before each experiment 1 mL of cell suspension containing 5 × 10^5^ cells was seeded into each well of a 24-well plate (Corning Inc., New York, NY, USA) and incubated at 37 °C with 5% CO_2_ for 24 h. Prior to infection, the culture medium was removed, and the epithelial monolayer was gently washed with pre-warmed PBS. Fresh DMEM supplemented with 5% FBS (1 mL per well) was then added before proceeding with infection assays.

### 2.7. Determination of Fungal Shedding by C. albicans Clinical Isolates

The determination of fungal shedding by *C. albicans* clinical isolates after VEC infection was performed as previously described (Protocol 2) [10]. Briefly, confluent monolayers of VEC were infected with the different *C. albicans* clinical isolates at a multiplicity of infection (MOI) of 1 (5 × 10^5^ CFU/mL) and incubated for 24 h at 37 °C in a humidified atmosphere containing 5% CO_2_. After incubation, supernatants were collected by gentle pipetting up and down ten times to recover both suspended fungal cells and those associated with exfoliated VEC and/or weakly adherent to the epithelial surface. The collected samples were centrifuged at 1500 rpm for 5 min using a Microfuge 18 centrifuge (Beckman Coulter, Brea, CA, USA), then the supernatants were discharged to eliminate free fungi, and the pellet further washed two times with pre-warmed DMEM supplemented with 5% fetal bovine serum (FBS).

The resulting pellets were treated with 0.2% Triton X-100 (Sigma-Aldrich, St. Louis, MO, USA) to lyse epithelial cells and release adherent fungal cells. Suspensions were then serially diluted and plated onto SDA plates, which were incubated at 37 °C. Colony-forming units (CFUs) were counted after 24–48 h of incubation.

### 2.8. Quantification of VEC Damage Induced by C. albicans Clinical Isolates

The level of cell damage induced by the *C. albicans* clinical isolates in VEC was evaluated after 24 h of infection, obtained with the same infection protocol described above, by a commercially available lactate dehydrogenase (LDH) release assay (Hoffmann-La Roche, Basel, Switzerland) according to the manufacturer’s instructions. Absorbance was measured spectrophotometrically at 492 nm with a reference wavelength of 620 nm. Vaginal epithelial cells damage was expressed as a percentage, calculated using a formula provided by the manufacturer. To determine cytotoxicity values, both negative and positive controls were included. Uninfected cells served as negative control, representing minimal LDH release, whereas maximal LDH release was obtained by complete lysis of uninfected cells with 1% Triton X-100.

### 2.9. Determination of Cytokine Production in Supernatants of VEC Infected with C. albicans Clinical Isolates and in the Vaginal Samples

Culture supernatants were collected from three independent experiments after 24 h of VEC infection at MOI of 1 with the different *C. albicans* clinical isolates. Samples were centrifuged to remove fungal cells and cellular debris and subsequently stored at −80 °C until analysis. Vaginal samples were collected from 19 women; however, due to insufficient material available from one sample, cytokine measurements were performed on 18 samples. Vaginal samples were diluted 1:10 in the appropriate assay buffer prior to cytokine quantification. The concentrations of IL-1α (PeproTech^®^, Thermo Fisher Scientific, Waltham, MA, USA), IL-1β (Invitrogen, Frederick, MO, USA) and IL-1 receptor antagonist (IL-1Ra) (Invitrogen, Frederick, MO, USA) were determined using enzyme-linked immunosorbent assay (ELISA) kits according to the manufacturers’ instructions.

### 2.10. Detection of Anti-C. albicans IgA Antibodies in Vaginal Samples by Indirect Immunofluorescence

For the detection of anti-*C. albicans* IgA antibodies, 20 µL of vaginal samples were analyzed using a commercial indirect immunofluorescence assay kit (GACTA, Vircell S.L., Granada, Spain). In the present study, the manufacturer’s protocol was modified by omitting the pre-absorption step with heat-inactivated *C. albicans* yeast cells. This modification was intentionally introduced to allow the detection of antibodies directed against both yeast-associated antigens and germ tube/hyphal antigens, rather than selectively restricting the analysis to germ tube-specific antibodies. To enable the detection of IgA antibodies in the vaginal samples, the assay protocol was further adapted by using a polyclonal goat anti-human IgA antibody conjugated to Alexa Fluor 555 (1.035 mg/mL; dilution 1:100; Jackson ImmunoResearch, Cambridgeshire, CB7 4EX, UK) as the secondary antibody. Fluorescently labeled fungal cells were visualized using an epifluorescence microscope (Nikon Eclipse 90i; Nikon Instruments, Tokyo, Japan) at 40× magnification. The scale bar corresponds to 10 µm. IgA reactivity was quantified by fluorescence image analysis using Fiji, an ImageJ2-based distribution (Version 2.14.0/1.54f; National Institutes of Health, Bethesda, MD, USA) [15]. Briefly, fluorescence images acquired with the TRICH filter set and saved in RGB format were split into individual channels, and the red channel corresponding to the Alexa Fluor 555 signal was selected. Background fluorescence was subtracted before image segmentation. Then, a fixed threshold (18–255) was applied to all images, which were then converted to binary format. The watershed function was used to separate adjacent fluorescent signals, and fluorescent particles were identified using the Analyze Particles tool (size range: of 20–80 px^2^; circularity range of 0.00–0.60). The resulting regions of interest (ROIs) were then applied to the corresponding original red-channel image, and the mean fluorescence intensity (MFI) was measured for each ROI.

### 2.11. Statistical Analysis

Statistical analyses were performed using GraphPad Prism software (version 10.6.1; San Diego, CA, USA). Data normality was assessed using the Shapiro–Wilk test, and data with a Gaussian distribution were analyzed using one-way Brown–Forsythe and Welch ANOVA, followed by Welch’s *t*-test for pairwise comparisons. For data that did not follow a normal distribution, the non-parametric Kruskal–Wallis followed by Uncorrected Dunn’s test were applied.

Associations between the different variables were assessed using Spearman’s rank correlation coefficient (*r*), and a correlation matrix was generated to summarize the correlation pattern among all parameters. Correlations were considered statistically significant if the *p*-value was ≤0.05.

## 3. Results

### 3.1. Description of the Cohort

A total of 25 *C. albicans* vaginal isolates were obtained from three different clinical groups as detailed in the Section 2, i.e., VVC/RVVC, Colonizing and Co-infections. The Co-infections group included patients with polymicrobial infections involving more than one bacterial species (e.g., *Escherichia coli* + *Streptococcus agalactiae*, *Ureaplasma urealyticum* + *Mycoplasma hominis*, *Ureaplasma parvum* + *Streptococcus agalactiae*), as well as patients with co-infections caused by a single bacterium (such as *Streptococcus agalactiae*, *Ureaplasma urealyticum*, or *Ureaplasma parvum*), as detailed in Table 1.

Although *C. albicans* isolates were obtained from 25 women, the respective vaginal samples were available only for 19 of them, including: six women with VVC/RVVC, four asymptomatic colonized carriers, and nine women with co-infections. These 19 samples were subjected to Next Generation Sequencing (NGS) analysis to characterize the vaginal microbiota, as described in the Section 2. The Community-State-Type (CST) classification was based on the predominance of specific *Lactobacillus* species, as described by Ravel et al. 2011 [16].

### 3.2. Vaginal Microbiota Description of the Study Cohort

**Figure 1:**
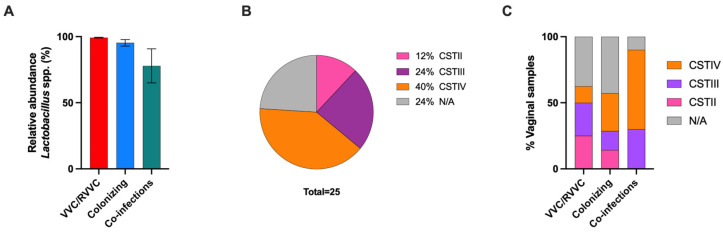
Community-State-Type (CST) distribution and microbial diversity across the study cohorts. (**A**) Relative abundance of the genus *Lactobacillus* spp. across the three cohorts: the graph shows the mean percentage ± SEM for each group; statistical analysis was performed by Kruskal–Wallis test. (**B**) Overall proportion of CST identified among all vaginal microbiome samples based on NGS analysis. N/A indicates data not available. (**C**) CST distribution among VVC/RVVC, Colonizing and Co-infections. N/A indicates samples not available.

The relative abundance of *Lactobacillus* spp. did not differ substantially among the three groups, although the study was not powered to exclude more subtle microbiome-associated differences. Co-infections samples showed a trend of lower *Lactobacillus* spp. abundance (Figure 1A). By analyzing the CST distribution in the whole study group, we showed that CST IV was the most prevalent, comprising 40% of the samples, followed by CST III (24%) and CST II (12%). The remaining 24% of the samples could not be categorized due to an insufficient sample amount for performing NGS analysis. Notably, none of the samples analyzed was classified as CST I (Figure 1B). Within the three patient cohorts, the distribution of CST appeared relatively similar; however, CST IV was prevalent among samples from the Colonizing group (28,6%) and in the Co-infections group (60%) (Figure 1C).

### 3.3. Evaluation of C. albicans Hyphae and PMN in Vaginal Samples from the Study Groups

**Figure 2:**
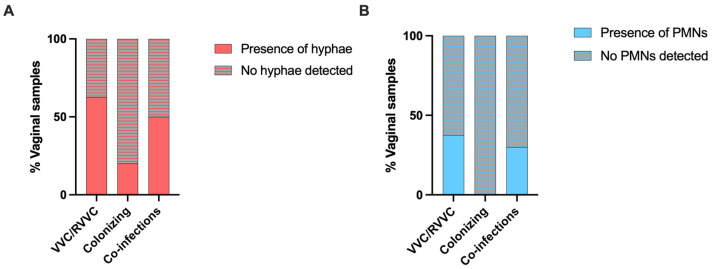
Percentage of *C. albicans* hyphae and PMN in vaginal samples from different clinical groups. (**A**) The red portion of the bar chart indicates the presence of hyphae. The red portion with horizontal stripes indicates the absence of detectable hyphae. (**B**) The cyan portion of the bar chart indicates the presence of PMN in the vaginal samples, whereas the cyan portion with horizontal stripes represents the absence of detectable PMN.

Vaginal samples were examined fresh immediately after sampling, and indicators of vaginal infection and inflammation, such as *C. albicans* hyphae and PMN presence, were evaluated by microscopic observation. As shown in Figure 2A, vaginal samples from VVC/RVVC had a higher percentage of hyphae (62.50%) compared to those from the Co-infections (50%) or Colonizing (20%) group. Moreover, PMN were observed only in samples from the VVC/RVVC (37.5%) and Co-infections (30%) group, whereas no PMN were observed in the Colonizing group (Figure 2B).

### 3.4. Determination of Cytokine Production and Anti-Fungal IgA Antibodies in Vaginal Samples

**Figure 3:**
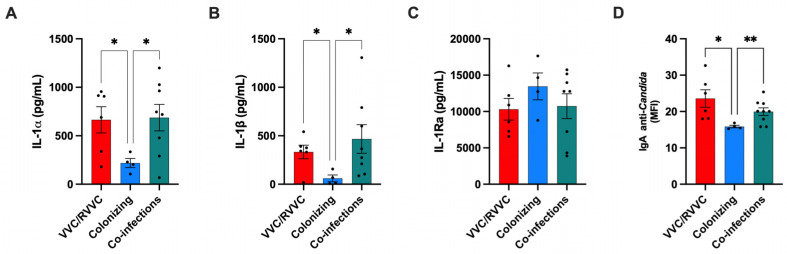
Inflammatory parameters in the vaginal samples of women in the VVC/RVVC, Colonizing, and Co-infections groups. (**A**–**C**) The graphs shown represent the mean pg/mL ± SEM of IL-1α, IL-1β, and IL-1Ra, respectively, in VVC/RVVC (red), Colonizing (blue), and Co-infections (green). (**D**) Anti-*Candida* IgA (MFI mean ± SEM) in vaginal samples across clinical groups (colors as above). Each dot represents an individual sample. Statistical significance was assessed by one-way Brown–Forsythe followed by Welch’s *t*-test. * *p* < 0.05; ** *p* < 0.01.

Cytokine levels in vaginal samples were measured in the different study groups. Although there is some variation in production levels among the different samples, our data shows a significantly greater amount of Interleukin (IL)-1α in the VVC/RVVC and Co-infections groups as compared to the Colonizing group (Figure 3A and Figure S1A). Similarly, IL-1β levels were variable between the different samples but overall, significantly higher in the samples from women from the VVC/RVVC and Co-infections groups, compared to the Colonizing group (Figure 3B and Figure S1B). In contrast, no significant differences were observed among the study groups in the production of IL-1Ra (Figure 3C and Figure S1C).

We also evaluated anti-fungal IgA antibodies in the vaginal samples. Despite inter-sample variability among groups, samples from the VVC/RVVC group exhibited significantly higher mean fluorescence intensity (MFI) values compared to the Colonizing group. Similarly, the Co-infections group showed significantly increased MFI values compared to the Colonizing group. No significant differences were observed between the VVC/RVVC and Co-infections groups (Figure 3D and Figure S1D).

### 3.5. Phenotypic Characterization of C. albicans Isolates In Vitro: Growth Kinetics and Biofilm Formation

**Figure 4:**
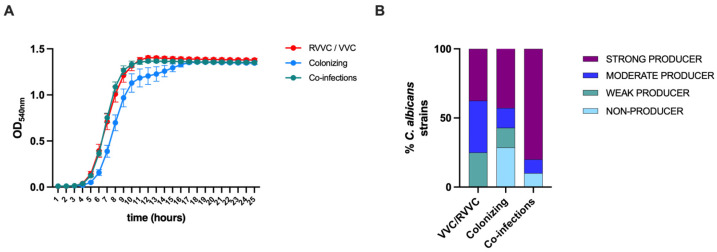
Growth kinetics and biofilm-producing phenotypes of *C. albicans* isolates. (**A**) Mean growth curves of *C. albicans* isolates obtained from VVC/RVVC (red), Colonizing (blue), and Co-infections (green) groups. (**B**) Percentage distribution of *C. albicans* isolates according to biofilm-forming capacity, categorized as strong (purple), moderate (blue), weak (green), or non-biofilm producers (cyan).

Growth curve analysis of *C. albicans* isolates revealed no significant differences among fungal strains derived from vaginal samples of VVC/RVVC, Colonizing or Co-infections (Figure 4A and Figure S2A). Indeed, all the *C. albicans* isolates exhibited largely overlapping growth profiles. A different distribution of biofilm-forming phenotypes was observed among clinical groups (Figure 4B and Figure S2B). *Candida albicans* isolates from the Co-infections group were predominantly classified as strong biofilm producers, whereas colonizing isolates displayed a more heterogeneous distribution, including a higher proportion of weak and non-biofilm producers. Isolates from the VVC/RVVC group were mainly represented by moderate or strong biofilm producers.

### 3.6. Determination of Fungal Shedding and VEC Damage In Vitro

**Figure 5:**
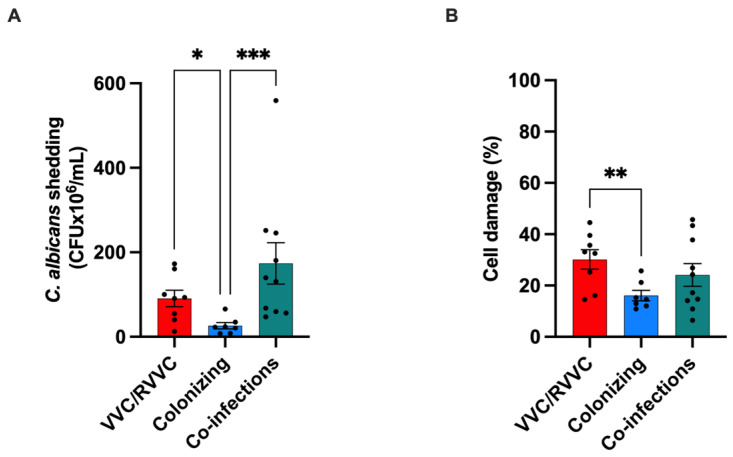
Fungal shedding and VEC damage. (**A**) Fungal shedding of *C. albicans* clinical isolates (VVC/RVVC: red; Colonizing: blue; Co-infections: green), expressed as mean ± SEM of CFU × 10^6^/mL. (**B**) Epithelial cell damage induced by *C. albicans* clinical isolates (colors as above), expressed as mean % ± SEM. Each dot represents the mean value of at least three independent experiments. Statistical analysis was performed using the Kruskal–Wallis test (**A**) or the one-way Brown–Forsythe followed by Welch’s *t*-test (**B**). Statistical significance is indicated as follows: *p* < 0.05 (*), *p* < 0.01 (**), *p* < 0.001 (***).

We next evaluated the capacity of the different *C. albicans* isolates to induce fungal shedding and VEC damage as previously described [10]. Our results show that fungal strains derived from both VVC/RVVC and Co-infections exhibited significantly higher fungal shedding compared to Colonizing fungal isolates. Notably, strains from Co-infections showed the highest level of fungal shedding among all groups (Figure 5A and Figure S3A). Moreover, VVC/RVVC fungal isolates caused significantly increased cell damage compared to Colonizing strains. A trend of increased cell damage was also observed for the Co-infections fungal strains, as compared to Colonizing strains, although without reaching statistical significance (Figure 5B and Figure S3B).

### 3.7. Cytokine Production in Supernatants of VEC Infected with C. albicans Vaginal Isolates

**Figure 6:**
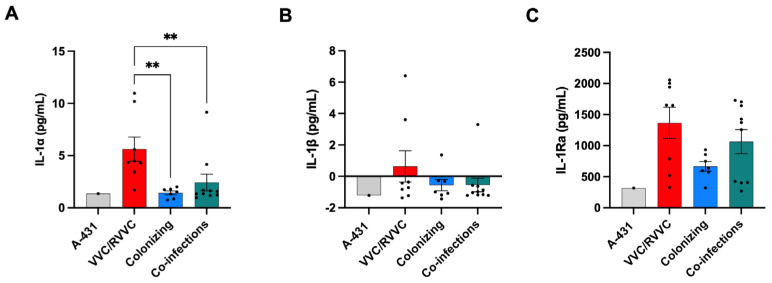
Cytokine production in supernatants of VEC (A-431 cell line) infected with *C. albicans* clinical isolates. IL-1α (**A**), IL-1β (**B**) and IL-1Ra (**C**) levels in supernatants of VEC uninfected (gray) or infected with *C. albicans* clinical isolates (VVC/RVVC: red; Colonizing: blue; Co-infections: green), are shown as mean ± SEM of pg/mL. Each dot represents the mean value of at least three independent experiments. Statistical analysis was performed using the Kruskal–Wallis test (**A**–**C**). Statistical significance is indicated as follows: *p* < 0.01 (**).

In order to compare the inflammatory profile observed in the vaginal samples with the cytokine response of infected VEC, the same cytokines were also evaluated in the supernatants of VEC infected with the *C. albicans* isolates obtained from the respective vaginal samples. Our data show that IL-1α production was significantly increased in supernatants of VEC infected with VVC/RVVC fungal isolates, as compared to supernatants of VEC infected with Colonizing and Co-infections isolates (Figure 6A and Figure S4A). Of note, although most of the supernatants were below the published detection limits, the mean of pg/mL of IL-1α was above the limit of detection only in the VVC/RVVC supernatants.

Moreover, no significant differences in IL-1β levels in supernatants obtained from VEC infected with *C. albicans* isolates belonging to VVC/RVVC, Colonizing, and Co-infections groups were observed, although, again, the only samples that exceed the kit’s detection limit are those included in the groups VVC/RVVC and Co-infections (Figure 6B and Figure S4B).

In contrast, high levels of IL-1Ra were detected across all the experimental conditions and no statistically significant differences were observed among the supernatants of VEC infected with *C. albicans* isolates belonging to all the different groups. However, a clear trend of increased IL-1Ra production was observed in supernatants of VEC infected with fungal strains belonging to the VVC/RVVC group and, to a lesser extent, to the Co-infections group as compared to the Colonizing group (Figure 6C and Figure S4C).

### 3.8. Correlation Analysis

**Figure 7:**
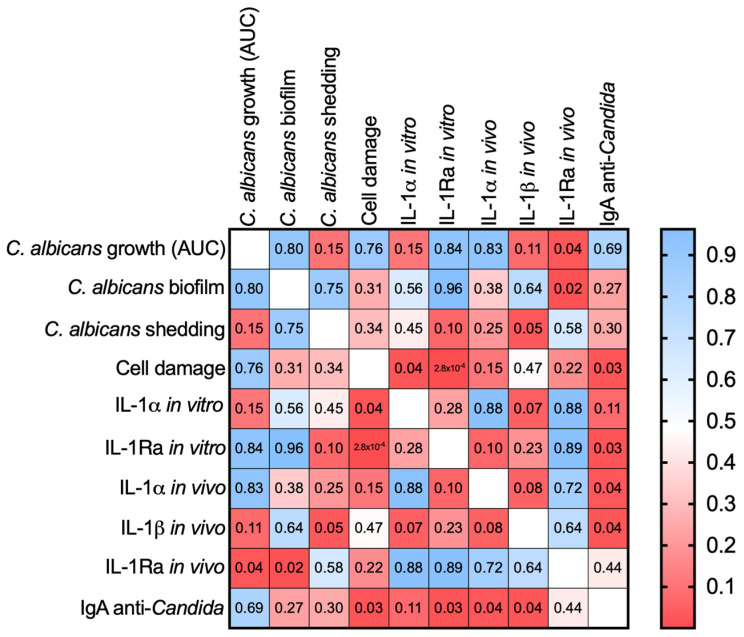
Correlation analyses. Spearman’s rank correlation analysis was used to evaluate associations between fungal traits (growth, biofilm formation, and shedding), epithelial cell damage, cytokine production (IL-1α, IL-1β, and IL-1Ra) measured *in vitro* and *in vivo*, and anti-*Candida* IgA in vaginal samples. The heatmap reports *p*-values for each pairwise comparison, with color intensity indicating significance (red, lower *p*-values; blue, higher *p*-values). Correlations were considered statistically significant at *p* ≤ 0.05.

To evaluate the relationship between the different parameters tested, Spearman’s rank correlation analysis was performed. The resulting *p*-value matrix is shown in Figure 7, while the corresponding matrix of correlation coefficients (r) is provided in the Supplementary Figure S5. Significant associations emerged between fungal phenotypic traits and host inflammatory responses. Both *C. albicans* growth and biofilm formation were significantly associated with IL-1Ra levels *in vivo* whereas *C. albicans* shedding showed a significant correlation with IL-1β levels *in vivo*.

Cell damage was significantly associated with IL-1α and IL-1Ra production *in vitro*, supporting a link between epithelial damage and cytokine release in the *in vitro* model. However, no significant correspondence was observed between the same cytokines measured *in vitro* and *in vivo*, suggesting that the *in vitro* and the *in vivo* settings capture complementary but non-overlapping aspects of the inflammatory response. Notably, anti-*Candida* IgA antibodies were significantly associated with cell damage, IL-1Ra levels *in vitro*, and IL-1α and IL-1β levels *in vivo*. Overall, these findings suggest that fungal phenotypic traits are mainly associated with *in vivo* inflammatory parameters, whereas the correlation between epithelial damage and anti-*Candida* IgA antibodies supports a link between mucosal antibody responses and local inflammatory pathways [17].

## 4. Discussion

In this study, we analyzed vaginal samples obtained from women with VVC/RVVC, who were asymptomatic colonized carriers, and who were co-infected. We assessed the vaginal microbiota composition by NGS and performed direct microscopic observation of vaginal wet mount slides to evaluate PMN infiltration and *C. albicans* morphology. In addition, we measured cytokine levels and anti-fungal IgA antibodies in the vaginal samples. In parallel, we characterized the corresponding *C. albicans* isolates *in vitro* by evaluating growth kinetics, biofilm-forming capacity, and their ability to induce fungal shedding, epithelial cell damage, and cytokine production in a VEC infection model. This combined analysis has allowed us to examine how fungal characteristics correlate to host responses in both clinical samples and experimental conditions.

By integrating microbiota profiling, inflammatory parameters in vaginal samples, and functional assays in a VEC infection model, our study provides insights into the strain-dependent and host-driven mechanisms underlying epithelial responses to *C. albicans* infection. Our findings suggest that the development of symptomatic vaginal fungal infection reflects a combination of strain-specific fungal properties and different hosts’ epithelial responses to specific fungal strains [18].

In the tested samples, vaginal microbiota analysis reveals a predominance of CST IV across the study cohort, particularly among asymptomatic colonized women and patients with co-infections. This observation is consistent with previous reports linking CST IV to reduced *Lactobacillus* spp. dominance and increased microbial diversity, a condition often associated with vaginal dysbiosis [16]. Notably, CST I, which is typically associated with a vaginal microbiota dominated by *L. crispatus* and characterized by high stability [16], has not been detected. This finding likely reflects the clinical nature of the study cohort, which included women with active VVC/RVVC, asymptomatic *C. albicans* colonization, or concurrent vaginal infections, rather than healthy low-risk individuals in whom CST I is more commonly observed. Additionally, the limited sample size may have reduced the chances of capturing CST I-associated profiles. Despite the CST distribution and relative abundance of *Lactobacillus* spp. not showing significant differences among clinical groups, these findings support the idea that VVC is not solely linked to dysbiosis but rather reflects a dysregulated host immune response to fungal colonization [19].

Further support for this immunopathological model has emerged from the microscopic analysis of vaginal samples. The presence of *C. albicans* hyphae and PMN has been observed only in patients with VVC/RVVC and co-infections, whereas asymptomatic carriers have shown minimal or no evidence of these features. This suggests that epithelial activation and PMN recruitment, rather than the mere presence of fungi, are the key drivers of disease manifestations [3,20].

Consistent with these findings, the analysis of cytokine levels in vaginal samples reveals a marked increase in pro-inflammatory cytokines IL-1α and IL-1β in women with VVC/RVVC and with co-infections. In contrast, IL-1Ra levels are comparably high across all groups, including asymptomatic carriers. These data suggest that IL-1Ra represents a broadly activated counter-regulatory response to fungal colonization, rather than a marker of disease severity. However, the concomitant persistence of elevated IL-1α and IL-1β despite high IL-1Ra levels indicates that this regulatory mechanism is insufficient to effectively restrain the inflammation. This imbalance supports the presence of a dysregulated immune response, which may contribute to the progression from asymptomatic colonization to symptomatic disease [4].

Moreover, we have observed an increase in anti-*C. albicans* IgA antibodies in vaginal samples from women with VVC/RVVC and in those with co-infections. Elevated anti-fungal IgA may indicate greater exposure to antigens, alterations in fungal morphology, or broader immune stimulation driven by bacterial co-pathogens. Notably, the presence of IgA does not seem to provide protection against inflammation, supporting previous evidence that antibody responses in VVC are not necessarily protective, but may instead indicate ongoing immune activation [3].

At the fungal level, our *in vitro* analyses do not show any significant difference in growth kinetics or biofilm-forming capacity among isolates from different clinical groups. This suggests that, at least for these parameters, the virulence of these fungal strains is not different in standard culture conditions where there is no interaction with host cells. These data are in line with our previous study, performed in a different cohort of vaginal samples, suggesting that *Candida* isolates from women with VVC or healthy colonized carriers do not differ in overall genetic profile or behavior in culture media (i.e., MLST profile, rate of growth, and filamentation), but they show strikingly different behaviors upon interaction with VEC [10].

According to our previous data [10], also in this new cohort of fungal vaginal isolates, functional assays *in vitro* in the VEC infection model reveal marked strain-dependent differences in fungal shedding and epithelial damage. Indeed, VVC/RVVC isolates induce significantly greater epithelial damage compared with colonizing strains, while both VVC/RVVC and Co-infections-derived fungal isolates promote the highest levels of fungal shedding. These findings suggest that the critical factor of *C. albicans* pathogenic potential is its ability to interact dynamically with VEC, rather than its growth properties [10].

Cytokine assessment in the VEC infection model further strengthens the hypothesis of strain-dependent differential cells activation in response to *C. albicans*. IL-1β has remained below the detection limit under all experimental conditions, consistent with limited inflammasome activation in this epithelial system. In contrast, IL-1α production has been selectively induced by VVC/RVVC isolates, the only strains capable of eliciting detectable levels of this pro-inflammatory mediator. Given the established role of IL-1α as an epithelial damage-associated signal, these findings strongly suggest that VVC/RVVC strains preferentially trigger epithelial stress responses that promote inflammation, regardless of the fungal burden [21]. In this context, IL-1α likely acts as an early alarmin released upon epithelial perturbation, that amplifies local inflammatory signaling and contributes to symptomatic disease [22]. This supports the concept that VVC onset is not solely dependent on fungal load or morphogenesis, but rather on the ability of specific “fungal strains determinants” to induce epithelial damage and dysregulated host responses, ultimately driving inflammation and clinical manifestations. Interestingly, the production of IL-1Ra is consistent across all conditions and it does not vary among different strain groups, which mirrors the observations made in vaginal samples. This suggests that the induction of IL-1Ra is a general vaginal epithelial response to *C. albicans* exposure, rather than a strain-specific protective mechanism. Importantly, the continued production of IL-1Ra, despite epithelial damage and the release of IL-1α, indicates that while anti-inflammatory regulatory pathways are activated, they are not sufficient to counteract the pro-inflammatory signals triggered by the interactions between pathogenic fungi and VEC [4]. To further integrate these observations, the correlation analysis provides additional insights into how fungal traits and host responses are interconnected across experimental settings. Notably, significant associations can be observed predominantly between fungal phenotypic features and cytokine levels measured *in vivo*, whereas only a limited number of associations are involved in *in vitro* readouts. *C. albicans* growth and biofilm formation are both associated with IL-1Ra levels *in vivo*, while fungal shedding is correlated to IL-1β *in vivo*. This pattern may suggest that the *in vivo* setting more closely reflects clinically relevant inflammatory responses than in simplified *in vitro* models. Consistent with this idea, cytokine responses measured *in vitro* show limited overlap with those observed in vaginal samples, with no significant associations detected between matched cytokines across *in vivo* or *in vitro* conditions; only a trend toward association has been observed between IL-1α *in vitro* and IL-1β levels *in vivo*. Similarly, IL-1Ra levels do not correlate between *in vitro* and *in vivo* conditions, further supporting the idea that regulatory pathways are differentially modulated depending on the biological context.

**Figure 8:**
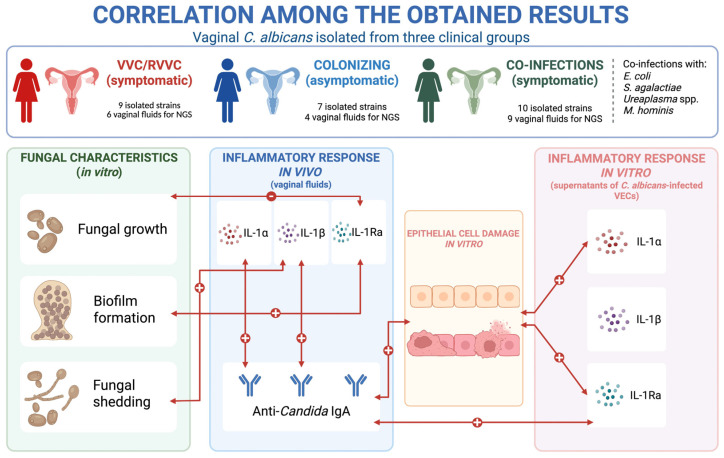
Overview of correlation analyses results. The phenotypic characteristics of *C. albicans* isolates (growth, biofilm formation, and shedding) are mainly associated with the *in vivo* inflammatory response of the patients. Epithelial cell damage induced by the *C. albicans* isolates is linked to the production of IL-1a and IL-1Ra *in vitro* and is associated with the local mucosal immune response (anti-*Candida* IgA). Red arrows with + or −: statistically significant (+: positive or −: negative correlations). Created in BioRender. Ardizzoni, A. (2026) https://BioRender.com/o65jx54 (accessed on 16 June 2026).

Interestingly, presence of anti-*Candida* IgA antibodies was associated with both epithelial damage and multiple cytokine responses, including IL-1Ra *in vitro* and IL-1α and IL-1β *in vivo*. This finding links mucosal antibody responses with local inflammatory activity, highlighting a close association between IgA antibodies, epithelial damage, and pro-inflammatory cytokine responses. A summary of the correlation among results obtained is depicted in Figure 8.

Our data support a multifactorial and immunopathological model of VVC, where inflammation is driven by specific strain properties of *C. albicans* combined with epithelial sensing mechanisms. In this model, pathogenic isolates cannot be distinguished by enhanced growth or biofilm formation capacity per se, but by their capacity to induce fungal shedding, epithelial damage and pro-inflammatory signaling [22]. A similar pro-inflammatory environment has been observed in vaginal samples from women with co-infections. This suggests that the presence of microbial co-infections may lower the threshold for epithelial activation or amplify pre-existing inflammatory responses. Rather than acting as direct causative agents, co-infecting microorganisms may modulate host susceptibility and contribute to inter-individual variability in disease expression [3,23]. From a clinical perspective, our findings may have important implications. The lack of a significant correlation between fungal shedding and epithelial damage emphasizes the limitations of therapeutic strategies that solely aim at reducing fungal load. Therefore, targeting epithelial inflammatory pathways, such as IL-1 signaling, or fungal factors that trigger epithelial immune response may represent more effective approaches for preventing symptomatic disease and recurrence. Overall, this study contributes to the growing body of evidence supporting host-directed and personalized strategies for managing VVC and its recurrence [19].

## 5. Conclusions

This study emphasizes the role of *C. albicans* strain-specific interaction with the host cells, possibly determining the clinical outcomes of VVC, regardless of the fungal load and biofilm-forming capacity. Indeed, in line with our previous results [10], and also in a new cohort of clinical samples, we have been able to characterize *C. albicans* isolates obtained from women with VVC/RVVC (and, although to a lesser extent, co-infections), based on their ability to cause fungal shedding, epithelial damage and trigger IL-1-driven inflammatory responses; as expected, the isolates obtained from healthy women in the Colonizing group, drive minimal epithelial activation.

Even though this study includes a relatively small sample size, particularly for vaginal fluid analyses, which limits the statistical power and the generalizability of the findings, further investigations will be carried out to include a larger number of samples.

Overall, these findings suggest the limitations of the current treatment strategies for VVC/RVVC that focus solely on eliminating the fungal infection. They also emphasize the need for host-directed therapies that target inflammatory pathways in the VEC and/or fungal factors that induce epithelial hyperactivation. Such strategies could provide more effective and lasting solutions for managing VVC and its recurrence.

## Figures and Tables

**Table 1:** Characterization of vaginal *C. albicans* clinical isolates from VVC/RVVC, Colonizing, and Co-infections groups, including fungal isolation, vaginal fluids availability, co-infecting bacteria, and Community-State-Types (CSTs).

	ID Strains	*C. albicans* Isolation	Vaginal Fluids	Other Microorganisms	CSTs
VVC/RVVC	2B	yes	yes	-	CSTIV
	5755	yes	-	-	-
	4	yes	-	-	-
	331	yes	yes	-	CSTII
	500	yes	yes	-	CSTIII
	5819	yes	yes	-	CSTIV
	975	yes	yes	-	CSTIII
	13844	yes	yes	-	CSTII
Colonizing	196	yes	yes	-	CSTIII
	197	yes	yes	-	CSTII
	G.Z.	yes	yes	-	CSTIV
	6188	yes	yes	-	CSTIV
	1	yes	-	-	-
	725	yes	-	-	-
	200	yes	-	-	-
Co-infections	A.C.	yes	yes	unidentified	CSTIV
	3045	yes	yes	unidentified	CSTIV
	13808	yes	yes	*E. coli* *S. agalactiae*	CSTIII
	13996	yes	yes	*S. agalactiae* *U. parvum*	CSTIII
	13838	yes	yes	*E. coli* *S. agalactiae*	CSTIII
	14045	yes	yes	*U. urealyticum* *M. hominis*	CSTIV
	2A	yes	yes	*U. parvum*	CSTIV
	5	yes	yes	*U. urealyticum*	CSTIV
	2C	yes	-	*S. agalactiae*	-
	929	yes	yes	*S. agalactiae*	CSTIV

## Data Availability

The original contributions presented in the study are included in the article; further inquiries can be directed to the corresponding authors. The NGS datasets analyzed in this study are available in the NCBI Sequence Read Archive (SRA) under BioProject accession number PRJNA1491074 (https://www.ncbi.nlm.nih.gov/bioproject/1491074) (accessed on 8 July 2026).

## Supplementary Materials

The following supporting information can be downloaded at https://www.mdpi.com/article/10.3390/jof12070509/s1. Figure S1: IL-1α (A), IL-1β (B), IL-1Ra (C) (all pg/mL), and anti-*Candida* IgA levels (MFI) (D), measured in each vaginal fluid sample from VVC/RVVC (red), Colonizing (blue), and Co-infections (green) groups.; Figure S2: (A) Growth curves of single *C. albicans* isolates: VVC/RVVC (red), colonizing (blue), and Co-infections (green). (B) Heatmap showing the biofilm-forming capacity of individual *C. albicans* isolates from each clinical group. Values represent the mean of at least three independent experiments performed for each strain. Figure S3: (A) *C. albicans* shedding from VVC/RVVC (red), Colonizing (blue) and Co-infections (green) strains, expressed as CFU × 10^6^/mL. (B) Mean ± SEM percentage of cell damage from VVC/RVVC (red), Colonizing (blue) and Co-infections (green) strains. For each strain three independent experiments were performed, and each dot represents a single independent experiment. Figure S4: (A) IL-1α, (B) IL-1β, and (C) IL-1Ra (all pg/mL). For each strain (VVC/RVVC: red; Colonizing: blue; Co-infections: green), three independent experiments were performed, and each dot represents a single independent experiment. Figure S5: Spearman’s rank correlation analysis was used to evaluate associations between fungal traits (growth, biofilm formation, and fungal shedding), epithelial cell damage, cytokine production (IL-1α, IL-1β, and IL-1Ra) measured *in vitro* and *in vivo*, and anti-*Candida* IgA in vaginal samples. The heatmap shows Spearman’s correlation coefficients (*r*) for each pairwise comparison, with color indicating the direction and strength of the correlation (red, negative; blue, positive; color intensity proportional to *r*).
