# Supplementary material for: Differential Virulence of Vaginal Candida albicans Isolates Correlates with Host Inflammatory Responses in VVC/RVVC
Source: J Fungi (Basel). 2026 Jul 10;12(7):509. doi: 10.3390/jof12070509 (PMC13413291; doi:10.3390/jof12070509)
Supplement: Supplementary file 1 [file jof-12-00509-s001.zip › jof-4411914-supplementary.pdf]

SUPPLEMENTARY FIGURES

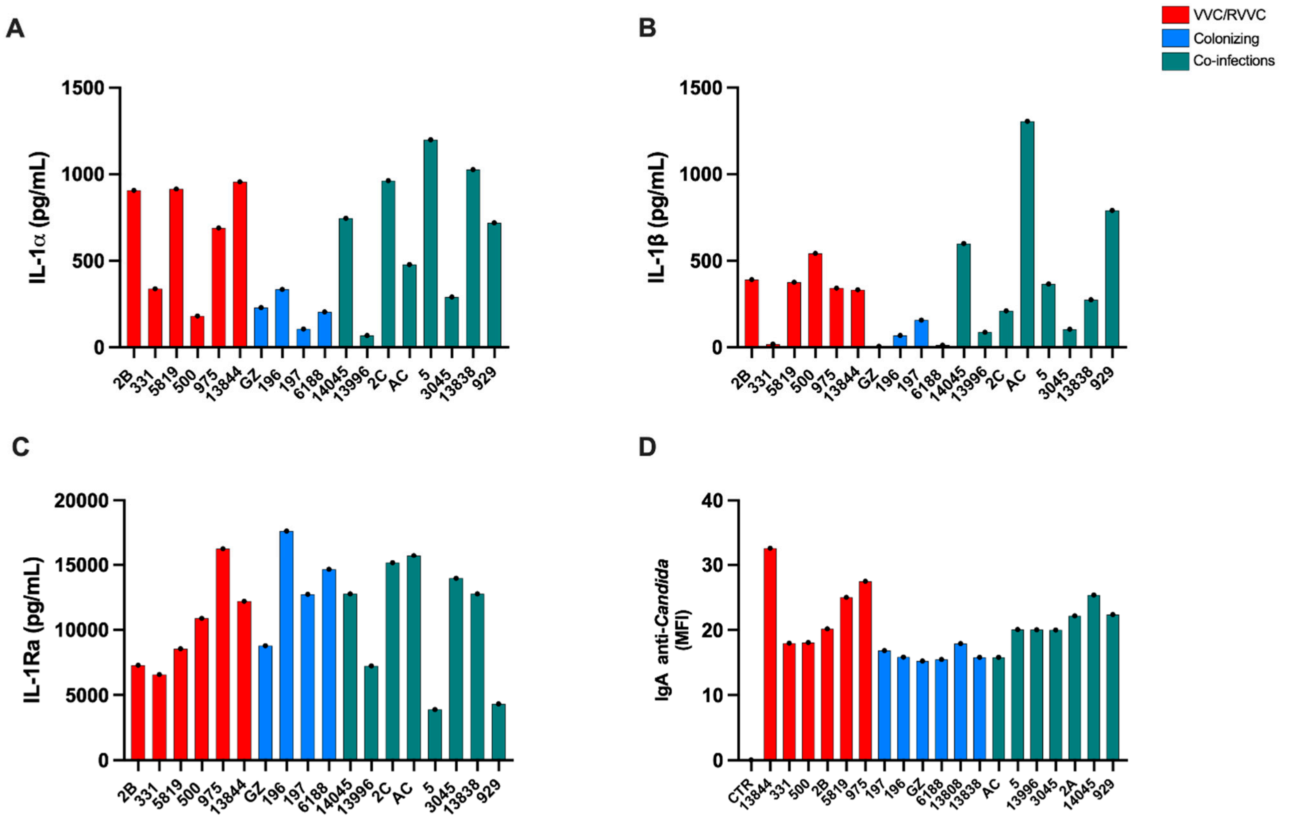

Supplementary Figure S1. IL-1α (**A**), IL-1β (**B**), IL-1Ra (**C**) (all pg/ml), and anti-*Candida* IgA levels (MFI) (**D**), measured in each vaginal fluid sample from VVC/RVVC (red), Colonizing (blue), and Co-infections (green) groups.

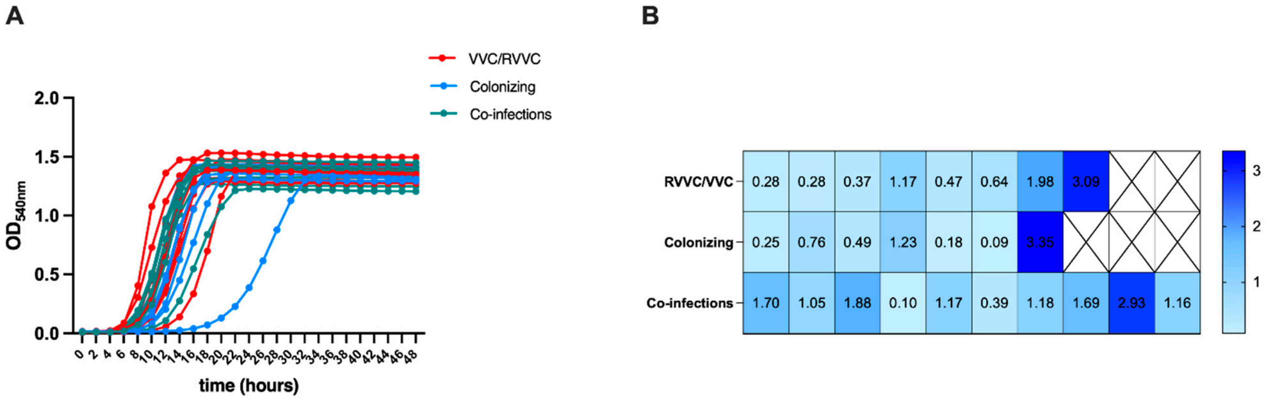

Supplementary Figure S2. (**A**) Growth curves of single *C. albicans* isolates: VVC/RVVC (red), colonizing (blue), and Co-infections (green). (**B**) Heatmap showing the biofilm-forming capacity of individual *C. albicans* isolates from each clinical group. Values represent the mean of at least three independent experiments performed for each strain.

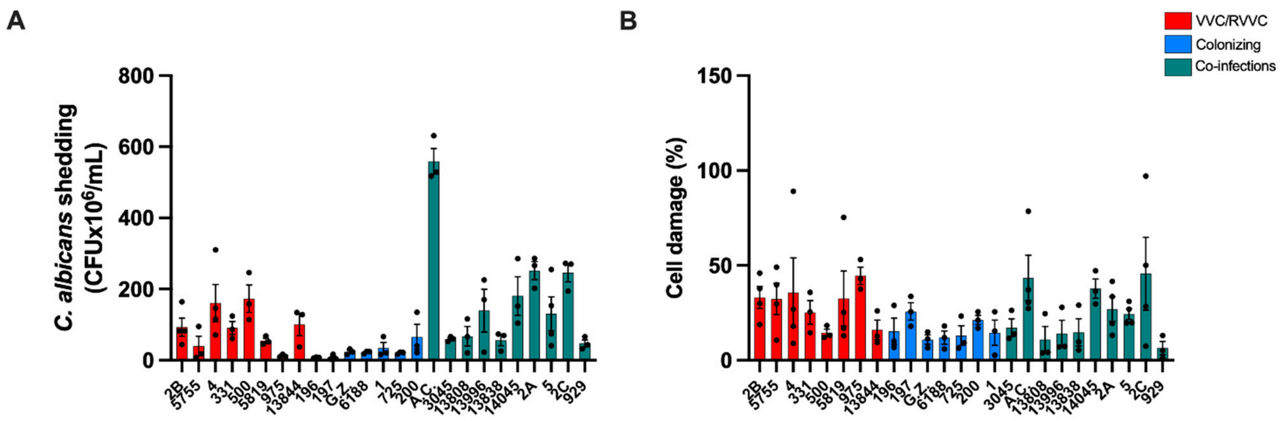

Supplementary Figure S3. (**A**) *C. albicans* shedding from VVC/RVVC (red), Colonizing (blue) and Co-infections (green) strains, expressed as CFU × 10^6/mL. (**B**) Mean ± SEM percentage of cell damage from VVC/RVVC (red), Colonizing (blue) and Co-infections (green) strains. For each strain three independent experiments were performed, and each dot represents a single independent experiment.

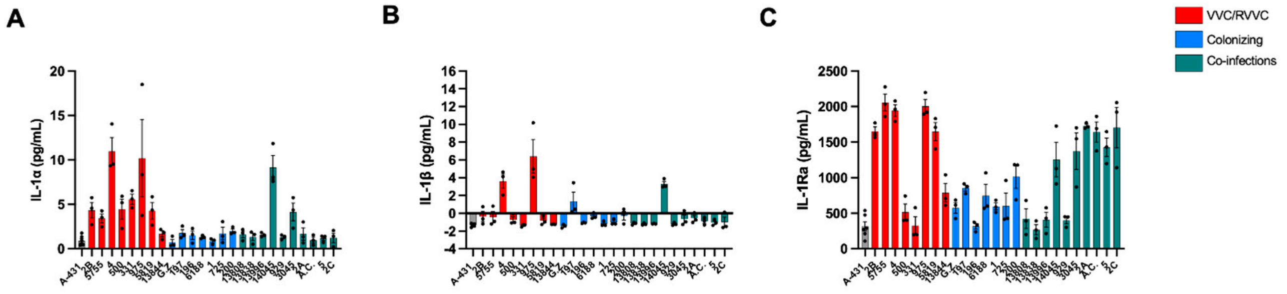

Supplementary Figure S4. (**A**) IL-1α, (**B**) IL-1β, and (**C**) IL-1Ra (all pg/mL). For each strain (VVC/RVVC: red; Colonizing: blue; Co-infections: green), three independent experiments were performed, and each dot represents a single independent experiment.

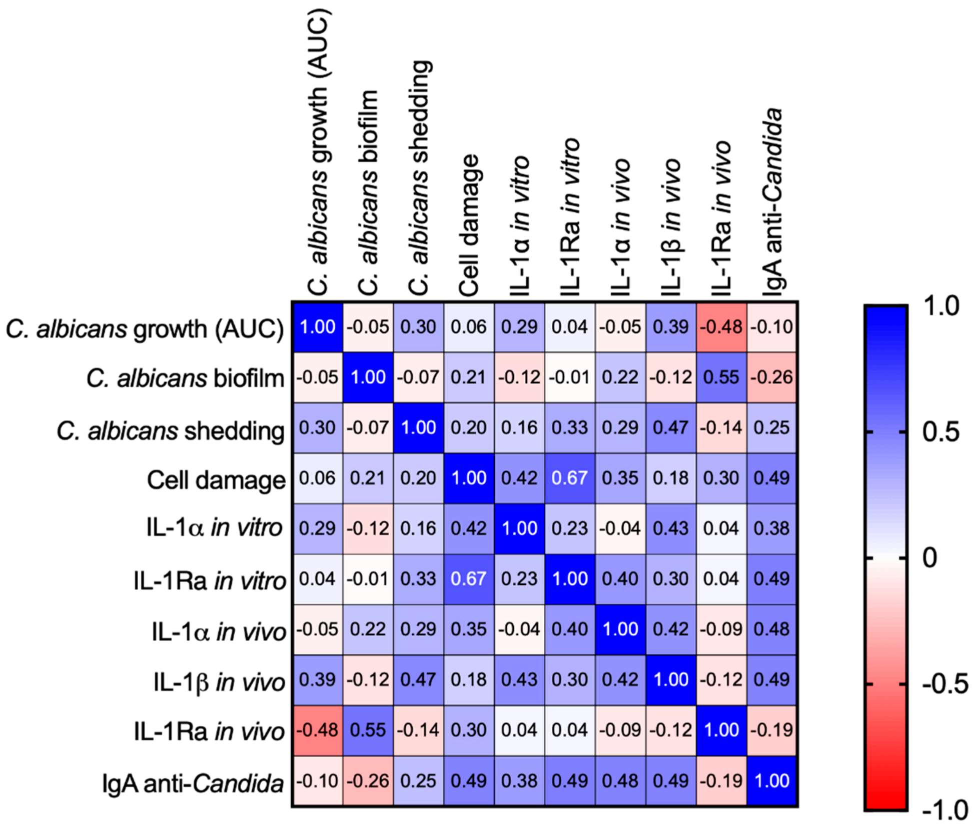

Supplementary Figure S5. Spearman's rank correlation analysis was used to evaluate associations between fungal traits (growth, biofilm formation, and fungal shedding), epithelial cell damage, cytokine production (IL-1α, IL-1β, and IL-1Ra) measured *in vitro* and *in vivo*, and anti-*Candida* IgA in vaginal samples. The heatmap shows Spearman's correlation coefficients (r) for each pairwise comparison, with color indicating the direction and strength of the correlation (red, negative; blue, positive; color intensity proportional to r).
